# 4140 Medical Students Perception of Plastic and Reconstructive Surgery and the Impact of Social Media Influencing their Opinion

**DOI:** 10.1017/cts.2020.217

**Published:** 2020-07-29

**Authors:** Fara Dayani, Paymon Rahgozar

**Affiliations:** 1 UCSF School of Medicine; 2 UCSF Department of Plastic and Reconstructive Surgery

## Abstract

OBJECTIVES/GOALS: The discipline of plastic and reconstructive surgery (PRS) is poorly understood by the public, primary care physicians, and nurses. The aim of our study is to assess medical students’ knowledge and perceptions of PRS as a discipline and explore factors influencing these opinions. METHODS/STUDY POPULATION: To assess medical student’s knowledge and perception of PRS, we distributed an online survey to all medical students at all training levels (i.e. first year to fourth year) enrolled at UCSF School of Medicine, San Francisco, CA during 2019-2020 academic year. In the survey, participants were asked to match 12 surgical subspecialties with 36 operative procedure scenarios. In addition, the survey included questions investigating the most common social medical platform used by medical students and the role of medical social media accounts in contributing to their knowledge of surgical subspecialties. RESULTS/ANTICIPATED RESULTS: Medical students demonstrated a profound gap in knowledge in plastic surgery. The majority of respondents correctly identified plastic surgeons as the primary surgeons performing the cosmetic procedures listed (abdominoplasty, facelift, and liposuction). PRS was identified as the primary specialty involved in breast reconstruction (94.4%) and burns surgery (88.9%). There was poor understanding of the role of plastic surgeons in hand surgery(16.6%), craniofacial surgery(14.8%), and head and neck cancer surgery(9.3%). 52.4% of respondents follow medical social media accounts and 45.6% of respondents indicated that social media contributed to their knowledge of surgical subspecialties. DISCUSSION/SIGNIFICANCE OF IMPACT: Medical students, who form the next generation of doctors, have limited knowledge regarding versatile applications of PRS. Misconceptions about the discipline of PRS negatively impacts resource allocation and hinders the delivery of care to patients that would profoundly benefit from this specialty. CONFLICT OF INTEREST DESCRIPTION: No authors have financial disclosures or conflicts of interest to declare.

